# Mental health risks of pandemic‐related media communication: The mediating roles of distinct types of perceived threat

**DOI:** 10.1111/risa.70079

**Published:** 2025-07-26

**Authors:** Sophia Schaller

**Affiliations:** ^1^ Institute of Media and Communication Science Technical University Ilmenau Ilmenau Thuringia Germany

**Keywords:** crisis risk communication, media exposure, mental health, perceived threat, public health crises

## Abstract

Research has shown that constant exposure to health crisis‐related information can negatively affect individuals’ mental health. Using data from a two‐wave panel survey of German citizens (*n* = 1162) conducted during the COVID‐19 pandemic, this study aims to examine whether and how the relationship between people's media exposure and mental health is mediated through distinct types of perceived threat. The results show that perceived threat posed by the virus (perceived health threat) and perceived threat regarding the consequences of governmental antipandemic measures (such as lockdowns) for personal freedom (perceived political threat) mediated media effects on depressive symptoms. However, the effects differed significantly depending on the type of information source. While more frequent exposure to high‐quality traditional news media (public broadcasters, national newspapers and magazines, and local and regional newspapers) positively affected depressive symptoms mediated by perceived health threat, the use of low‐quality traditional news media (private broadcasters and tabloids) and social media platforms did this mediated by perceived political threat. By providing a nuanced account of the relationship between media exposure, perceived threat, and mental health during times of a major health crisis, this study offers practical insights into how harmful effects of health crisis risk communication could be mitigated.

## 1 INTRODUCTION

In recent years, public health crises, such as epidemics and pandemics, have increasingly unsettled large regions around the globe (Chon & Park, 2019). By definition, a crisis occurs when a certain risk becomes manifested and exceeds public response efficacy (Heath & O'Hair, 2010). Notably, recent as well as current crises (e.g., climate change and armed conflicts) often pose a variety of risks to individuals and society at the same time, and this also applies to public health crises. Previous epidemics and pandemics resulted in major disruptions of health, political, and economic systems. Moreover, they presented numerous significant life stressors for individuals worldwide (Leung et al., 2022).

In order to successfully overcome such challenging times, crisis communication through the media—that is, mediated risk communication in the face of an (health) emergency situation (Lundgren & McMakin, 2018)—plays a vital role (Sellnow & Seeger, 2021). As a central source of information, the media often covers public health crises extensively over weeks, months, or even years (Hase & Engelke, 2022; Rossmann et al., 2018) to keep the citizens up‐to‐date about the ongoing situation (Sellnow & Seeger, 2021). By reporting on threats and protective measures and relying on negative emotional appeals (e.g., fear appeals) to alert the public, the media further can serve a significant function in encouraging citizens to engage in protective behaviors (Hase & Engelke, 2022; So et al., 2016).

However, repeated exposure to such threatening, often emotionally arousing crisis‐related content in the media can deteriorate individuals’ mental health (Chu et al., 2022; Houston et al., 2018). For instance, during the COVID‐19 pandemic as the most recent global health crisis, stress‐related mental health problems (e.g., depressive symptoms) increased significantly worldwide (Robinson et al., 2022), and there is much evidence that a frequent crisis‐related media exposure contributed to these developments (Chu et al., 2022). In addition to these rather immediate detrimental consequences, distressing experiences following crisis‐related media exposure can influence individuals’ responses to upcoming crises (Thompson et al., 2019) and thus may affect future crisis management and overall social functioning. Therefore, it is crucial for risk communication to understand the underlying psychological mechanisms through which the media influences recipient's mental health during times of health crisis in order to mitigate such unintended negative impacts in the future.

The application of the differential susceptibility to media effects model (DSMM) to disasters (Houston et al., 2018) generally suggests that media exposure indirectly affects mental health by shaping people's cognitive and emotional responses to health crises. Drawing further on the transactional stress model (TSM; e.g., Lazarus, 1999) and literature from the field of health risk communication (e.g., So, 2013), the media's influence on individuals’ crisis‐related *perceived threats* may be a particularly important mechanism in explaining mental health risks. Against this backdrop, the current study aims to gain insights into how perceived threats mediate the effects of media consumption on mental health during a major health crisis.

Using data from a two‐wave panel survey conducted during the COVID‐19 pandemic, this study thereby acknowledges the multifaced nature of pandemic threat and related media coverage, which has mostly been neglected in existing research on public health crisis risk perceptions and communication (e.g., B. Chan et al., 2024). Specifically, the present research examines the mediating roles of both perceived health threat and perceived political threat to personal freedom due to governmental antipandemic measures. Furthermore, this study considers potential effects from three types of information sources, namely, high‐quality traditional news media (public broadcasters and quality newspapers and news magazines), low‐quality traditional news media (private broadcasters and tabloids), and social media platforms. Although these information sources differ in the ways in which public health crises are communicated and discussed (e.g., extent of emotionalization, Rossmann et al., 2018) and may therefore vary in their influence on recipients’ mental health and perceived threats, research on possible differential effects is still lacking in this context. Finally, unlike prior studies (e.g., Wu et al., 2021), this research examines mental health‐related media effects not at the initial stage but concentrates on the maintenance phase of the COVID‐19 pandemic. This extension of existing literature to later stages is important, because phase models (Reynolds & Seeger, 2005) suggest that risk communication on health crisis‐related threats by the traditional news media (Hase & Engelke, 2022) and on social media platforms (Jin & Spence, 2021) changes across different stages. Moreover, individual (de)sensitization to these contents over time may be possible as well (Thompson et al., 2019), thus raising the question of whether prior findings on mental health‐related media effects also hold true for a later phase of health crises. Therefore, overall, the present study contributes to both their identification and a more comprehensive understanding of mental health risks associated with risk communication during public health crises.

## 2 MEDIA EFFECTS ON MENTAL HEALTH DURING PUBLIC HEALTH CRISES

In times of crisis, media use mostly increases and people turn to multiple information sources to stay informed about ongoing events (Liu et al., 2016; Nielson et al., 2021), with traditional news media (including their online websites and apps) and social media platforms being the preferred sources during public health crises (Nielson et al., 2021). Theories on information‐seeking behaviors, such as the risk information seeking and processing model (Griffin et al., 1999), the planned risk information seeking model (Kahlor, 2010), and the uncertainty management theory (Brashers, 2001), explain this by referring to people's increased need for information during such extreme risk situations: Individuals try to cope with emerging feelings of threat, uncertainty, and ambiguity by extensively seeking information.

Although crisis‐related risk information provided by the media has been shown to help individuals make sense of complex situations (Melki et al., 2022), a substantial body of research shows that repeated exposure to such content can backfire and have detrimental consequences for mental health: Previous studies across various crises—including natural disasters (Houston et al., 2018), terrorist attacks (Pfefferbaum et al., 2021), and, most importantly, epidemics (Thompson et al., 2017) and recently the COVID‐19 pandemic (Chu et al., 2022)—have demonstrated that higher media use negatively affects several stress‐related mental health problems, such as depression or posttraumatic stress disorder. In the context of health crises, the nonstopping news cycle, the massive amount of information, as well as the emotionally arousing nature of related content are considered reasons for the negative impact of media exposure on mental health (Chu et al., 2022; Houston et al., 2018). In particular, such content is characterized by a high amount of uncertainty, negative emotional appeals, and an overemphasis on threats and losses at the expense of (effective) solutions (Hase & Engelke, 2022; Klemm et al., 2016), all of which may trigger distressing responses (Dolinšek et al., 2024).

There are different theoretical approaches providing explanations for *how* the relation between crisis‐related media exposure and mental distress may unfold, which could also be helpful in the context of public health crises. From a media effects theory perspective, the DSMM (Valkenburg & Peter, 2013)—which synthesizes theories and empirical evidence from various fields of media effects research—and its application to disasters (Houston et al., 2018) provide important indications. Specifically, the DSMM proposes that media effects on psychological outcomes, such as mental health or distress, are *indirect* (i.e., second‐order media effects) mediated by first‐order effects of media exposure on individuals’ cognitive and emotional responses. Houston et al. (2018) demonstrated that this sequential process applies to disaster situations (natural and human‐caused), suggesting that it also extends to public health crises. Thus, media exposure may have an indirect effect on mental distress that is mediated by recipients’ health crisis‐related responses.

Psychological literature offers deeper insights regarding the question of which *precise* responses might be possible mediators: In particular, according to the TSM (Lazarus, 1999; Lazarus & Folkman, 1984), mental distress (e.g., depressive symptoms) is elicited by stressful *threat perceptions*. Threat perceptions occur when individuals assess the consequences of a negative event jeopardizing basic human needs, such as health, personal freedom, or social connection (Karademas et al., 2008; Lazarus, 1999; So, 2013). The TSM (e.g., Lazarus, 1999) and related concepts of risk and threat (e.g., So, 2013) suggest that perceiving a basic need to be threatened comprises negative cognitive appraisals and negative emotional responses, as both are highly and reciprocally related. Crucial cognitive appraisals are perceived severity and perceived susceptibility (Lazarus, 1999; So, 2013). Perceived severity describes as to how serious individuals generally perceive the threat's consequences, while perceived susceptibility refers to the perception of personal threat and individual loss (So, 2013; So et al., 2016). The emotional component varies depending on the specific type of threat (Lazarus, 1999; So, 2013). For example, the feeling of fear arises when individuals perceive a serious threat to physical well‐being, whereas the feeling of anger is the corresponding emotion of perceiving an event to be a demeaning offense (So, 2013), such as when one considers that an event jeopardizes his or her personal freedom.

Accordingly, the TSM proposes that mental distress results from an individual process in which people appraise a potential stressor as threatening and respond with corresponding, threat‐related emotions (Biggs et al., 2017; Lazarus, 1999). Importantly, such distressing threat perceptions especially occur in situations where coping appraisals are typically low (Brose et al., 2021; Lazarus & Folkman, 1984; Wang et al., 2021). That is, in situations where people feel that few or no actions can sufficiently control a threatening stressor (low response efficacy) and/or believe they are incapable of taking such actions (low self‐efficacy; Lazarus, 1999; So, 2013)—as is often the case during a public health crisis with its numerous uncontrollable threats (Brose et al., 2021). Given that (health) crises can be experienced not only directly but also indirectly through the media, scholars argue that related media exposure can represent a significant life stressor intensifying recipients’ threat perceptions during such times (Stainback et al., 2020; Thompson et al., 2017). So, overall, media and psychological theories suggest that an ongoing media exposure to information on health crises repeatedly activates cognitive‐emotional stress responses and threat perceptions in particular, which finally result in mental distress.

### 2.1 The mediating role of health crisis‐related perceived threats

There is compelling evidence that, during a public health crisis, risk communication through traditional news media and on social media platforms shapes people's perceived *health* threat from the respective virus (B. Chan et al., 2024; M.‐P. Chan et al., 2018; Friemel & Geber, 2023; Garfin et al., 2022; Schaller et al., 2024; Seo, 2021). Problematically, while frequent exposure to health crisis‐related media information can increase perceived health threat and thereby promote protective behaviors (e.g., Friemel & Geber, 2023), health threat perception also mediates the negative effects of media use on individuals’ mental health. More specifically, previous studies on the COVID‐19 pandemic have shown that (social) media exposure was positively associated with people's perceived health threat, which in turn was related to stress‐related mental health problems, such as depressive symptoms (Majeed et al., 2020; Olagoke et al., 2020; Wang et al., 2021; Wu et al., 2021).

The literature on health risk communication (e.g., extension of the extended parallel process model [E‐EPPM]; So, 2013) suggests that mediated communication influences health threat perceptions by presenting fear‐inducing threat‐related content and coping information (e.g., on protective measures). If coping information is insufficient or even absent, exposure to fear‐inducing health threat messages can have adverse effects on individuals (Friemel & Geber, 2023; So, 2013). Accordingly, the distressing mediation effects of perceived pandemic health threat observed by prior research (e.g., Olagoke et al., 2020) may be due to the media frequently covering and discussing crisis‐related threats in an emotionally arousing manner and tending to focus less on how to deal with such threats (Hase & Engelke, 2022; Klemm et al., 2016).

However, public health crises (e.g., epidemics and the COVID‐19 pandemic) do not only pose health‐related threats. Rather, they present a multifaceted type of threat exposure. Accordingly, the media portray medical issues, such as infection rates and deaths, as well as serious political, economic, and social issues (Hase & Engelke, 2022; Ophir & Jamieson, 2020). Still, unlike perceived health threats, such types of threat exposure and perceptions have been largely unexplored in extant risk communication research on public health crises (e.g., B. Chan et al., 2024; Chu et al., 2022). And, in consequence, the question of how the media affects different health crisis‐related threat perceptions remains unanswered—despite its potential consequences for individuals’ mental health.

During the COVID‐19 pandemic, perceived threat to personal freedom from governmental antipandemic measures, that is, political threat perception, became particularly prominent (Magson et al., 2021; Schaller et al., 2024). Although the threat arising from the virus itself dominated the media coverage, the impact of governmental restrictions on peoples’ everyday lives and their threat for personal freedom were also extensively discussed (Hase & Engelke, 2022; Reinemann et al., 2024). Moreover, not only perceived health threat (e.g., Wang et al., 2021) but also perceived threat to personal freedom may have negatively affected individuals’ mental health (Magson et al., 2021), making a mediating effect of perceived political threat likely as well. Therefore, focusing exclusively on the mediating role of perceived health threat to understand the relationship between pandemic‐related media use and mental health may be inadequate.

Thus, this study concentrates on both and, guided by Lazarus (e.g., 1999) and related literature (So, 2013), conceptualizes *perceived health threat* as encompassing the appraisal of the physical danger of the coronavirus and the related emotion of fear. *Political threat perception* reflects the appraisal of threat to fundamental rights, particularly personal freedom, due to governmental antipandemic measures and the corresponding emotion of anger (Ball & Wozniak, 2022).

### 2.2 Differential media effects of distinct types of information sources

To uncover (indirect) media effects on mental health during health crises, it is crucial to consider that they could vary depending on the information sources people use, as various traditional news media and social media platforms differ in how they communicate such events.

#### 2.2.1 Differences between distinct types of traditional news media

Usually, crisis coverage by traditional news media (including their online websites and apps) is guided by routines and standards (Olsson & Nord, 2015). Nevertheless, distinct patterns of crisis risk reporting can often be observed among different types of news media (Jacobs et al., 2016; Rossmann et al., 2018; Wiedicke et al., 2024). Several European countries (e.g., Germany, Italy, and Poland) have dual broadcasting systems with public‐ or governmental‐funded channels alongside private, commercial ones (Arbaoui et al., 2020). For example, in Germany, public broadcasters (e.g., *ARD*) are largely funded by public fees and are therefore legally required to fulfill specific needs, such as enabling opinion formation by providing diverse information (Bundeszentrale für politische Bildung, 2024). In contrast, private broadcasters (e.g., *RTL*), reliant on advertising, are more market‐driven and do not have a legal programming mandate. Therefore, their news coverage tends to be more sensational—that is, more focused on emotionally arousing topics and reporting styles to attract audiences—less balanced, and thus of lower overall quality than public media coverage (Arbaoui et al., 2020; Jacobs et al., 2016). Similar differences also exist in the press of various European countries: While popular tabloids (e.g., *Bild* in Germany or *The Sun* in the United Kingdom) concentrate on sensationalism and entertainment (Magin, 2019), the quality press stands for “more serious and objective coverage of political, economic, scientific, and cultural news” (Rossmann et al., 2018, p. 6). Taken together, scholars (e.g., Viehmann et al., 2021) distinguish between more sensational *low‐quality traditional news media*—that is, commercial broadcasting services and tabloid press—and *high*‐*quality traditional news media*—that is, public broadcasting services and quality newspapers and news magazines—which generally report in a less sensational manner and present more balanced information.

Such differences in sensational content and reporting style have also been shown for news media coverage of different public health crises. For instance, a content analysis of European media coverage of the H1N1 pandemic indicated that high‐quality press dramatized the health threat less and provided more information on protective measures than tabloid media (Rossmann et al., 2018). Moreover, in the context of COVID‐19‐related news media coverage, Hase and Engelke (2022) found that headlines in UK tabloids were more fear‐inducing than those in high‐quality newspapers. Similarly, Ogbodo et al. (2020) observed that UK high‐quality news media used a more hopeful tone than tabloids. In Germany, a content analysis of television news (Maurer et al., 2021) also found differences in news aspects between high‐ and low‐quality media, with commercial broadcasters focusing more on social issues, such as social life during the pandemic, than public broadcasters. Therefore, it can be assumed that low‐ and high‐quality news media portray health crises differently—both in the extent to which they report on specific threats and in how sensationalized and emotionally charged their communication is. In consequence, exposure to these different types of news media may vary in its influence on perceived threats and mental health.

While studies on mental health in this context are lacking, previous survey‐based research indicates that low‐quality news media coverage of a public health crisis affects individuals’ perceptions and attitudes differently from high‐quality news media coverage. For example, a study from Germany (Wiedicke et al., 2024), conducted at the beginning of the COVID‐19 pandemic, found that people who primarily watched private television news perceived the health threat of the virus to be more serious than those relying on public television news. Moreover, Viehmann et al. (2021) showed that using high‐quality news media reduced the perception of a dramatized public discourse about the COVID‐19 pandemic among German citizens, whereas using low‐quality news media did not. Finally, Frissen et al. (2020) found that Belgian residents who primarily relied on low‐quality tabloid media, in comparison to those predominately using high‐quality public media, were less in favor of the measures taken by the government to contain the disease. Thus, they may have been more concerned about these measures and its consequences for personal freedom than recipients of high‐quality news media. Overall, this suggests that exposure to low‐quality news media coverage of a public health crisis may be more distressing for individuals. So, the following hypothesis is proposed for distinct media effects on mental distress:

H 1 Exposure to COVID‐19‐related information through low‐quality traditional news media has a stronger positive effect on recipients’ mental distress than high‐quality traditional news media exposure.

Moreover, previous research suggests that these potentially differential media effects are mediated by perceived health and political threat. Therefore, it is hypothesized:

H 2 The supposed effects of exposure to COVID‐19‐related information through low‐quality and high‐quality news media on recipients’ mental distress are mediated by individuals’ perceived health and political threat.

#### 2.2.2 Differences between social media platforms and traditional news media

Along with traditional news media, social media platforms (e.g., Instagram, YouTube, and Facebook) have increasingly become important sources not only to seek but also to share and exchange information in crisis situations (Chon & Park, 2019; Liu et al., 2016), including public health crises (Nielson et al., 2021). However, crisis risk communication on social media does not conform to the journalistic standards and routines of established news media: User‐generated content shared on social media platforms is rarely subject to quality control and frequently unverified, which can lead to misinformation and finally misperceptions of threat (Lee et al., 2023; Sell et al., 2020). Moreover, research has shown that, during public health crises, citizens use social media platforms to express their perceived threats and related negative emotions, such as fear and anger (Iglesias‐Sánchez et al., 2020; Song et al., 2017; Wahidie et al., 2021). For instance, a qualitative content analysis of German COVID‐19‐related posts on Facebook, Twitter, and YouTube found that concerns about governmental restrictions and their consequences for personal freedom were shared and discussed frequently (Wahidie et al., 2021). On the one hand, seeing such content may help users cope with their own perceived threats and offer social support during critical times (Neubaum et al., 2014). On the other hand, however, encountering negative emotional expressions embedded in engaging personal stories on social media platforms may be more detrimental to mental health than receiving information from traditional news media (Oh et al., 2021; Valkenburg et al., 2016)—especially when compared to receiving information from high‐quality news media, which tend to present threats in a less emotionally arousing manner (Rossmann et al., 2018).

Prior survey‐based research has produced mixed results on the role of social media platforms during public health crises. Several studies reported that social media exposure increased individuals’ perceived health threat (e.g., Luo et al., 2021) and adverse psychological reactions (Chu et al., 2022) more than exposure to traditional media. Other studies found no differences (e.g., Riehm et al., 2020) or even reported that traditional media use had more harmful effects on perceived health threats (e.g., Entradas, 2022) and mental health (e.g., Neill et al., 2023). However, none of these studies examined whether the effects of using social media differ from the effects of *distinct* types of traditional news media (i.e., low‐ and high‐quality), which could be one reason for their contradictory findings. Thus, whether—and if so, how—the impact of social media exposure on distinct types of perceived threat and recipients’ mental health differs from the effects of using high‐quality and/or low‐quality news media still remains unclear. Therefore, the following research question (RQ1) was formulated:

RQ 1 *How do the potential direct and indirect effects of exposure to COVID‐19‐related information through social media platforms differ from the effects of exposure to COVID‐19‐related information provided by high‐quality and low‐quality traditional news media?*

Finally, an additional limitation of prior research (e.g., M.‐P. Chan et al., 2018; Chu et al., 2022; Wu et al., 2021) lies in its focus on examining mental health‐related media effects solely at the initial stage of risk communication on a public health crisis. However, phase models of risk and crisis communication (e.g., Reynolds & Seeger, 2005) suggest at least three different stages: After the initial phase, the maintenance phase unfolds, in which this study is particularly interested, preceding the resolution phase. The maintenance phase of a (health) crisis may be associated with distinct communication patterns in the media (Hase & Engelke, 2022; Jin & Spence, 2021; Reinemann et al., 2024) as well as distinct information‐seeking behaviors (Dan & Brosius, 2021; Schaller et al., 2024) compared to the initial stage. Also, individual (de)sensitization to distressing media content may be conceivable over the course of a health crisis (Thompson et al., 2019), potentially resulting in different media effects on mental health and perceived threat during the maintenance phase. Therefore, more research is needed on later stages of public health crises.

## 3 METHODS

### 3.1 Procedure and sample

**FIGURE A1:**
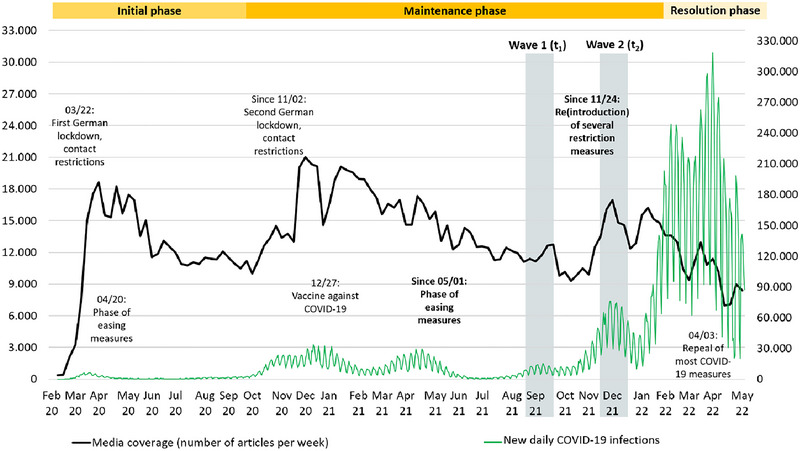
The figure illustrates the German developments of new daily COVID‐19 infections, related antipandemic governmental measures, and media coverage, based on Schaller et al. (2024). Media coverage data were collected from the LexisNexis database by using the search terms “corona” and “covid,” while infection data were obtained from the Robert Koch Institute (2023). In Germany, various antipandemic governmental measures were introduced to contain the rapid spread of COVID‐19. These included lockdowns (temporary closures of public venues such as restaurants, shops, schools, universities, offices, and cultural institutions), travel restrictions, limits on social gatherings, and various hygiene regulations such as mandatory face masks in public spaces, COVID‐19 testing, and quarantine requirements in case of coronavirus infection. With regards to the specific time periods of this study, during *t*_1_ (September 15–23, 2021), the number of new daily COVID‐19 infections was relatively low, and accordingly, most antipandemic governmental measures were absent. In contrast, *t*_2_ (December 9–20, 2021) coincided with Germany's fourth COVID‐19 infection wave, peaking at around 70,000 new daily infections. During this time, several antipandemic measures were therefore in force, including the limitation of gatherings to a small number of people, mandatory remote work where possible, COVID‐19 testing requirements for unvaccinated individuals, and compulsory face masks for all school pupils. This more serious situation is also reflected in the volume of news media coverage, which was higher during *t*_2_ than during the first survey period, aligning with findings from other content analyses (e.g., Reinemann et al., 2024).

The relationships between media exposure, perceived threats, and mental distress were examined using data from two waves of a quota‐stratified panel survey of German citizens implemented by the online access panel respondi. The first wave (*t*_1_, *n* = 1344) was conducted in September 2021, when the COVID‐19 infection rate in Germany was relatively low and most governmental social restriction measures had been lifted. The second wave (*t*_2_, *n* = 1162) was in December 2021, and coincided with the fourth COVID‐19 infection wave in Germany. To combat the rapid spread of the virus, several governmental antipandemic measures (though no full lockdown as during the pandemic's initial stage) were (re)introduced shortly before *t*_2_—such as restrictions of gatherings to a small number of people and COVID‐19 testing requirements for unvaccinated employees (for more details, see Appendix Figure A1). Thus, the time frame of this study falls into the maintenance phase of the COVID‐19 pandemic (Reynolds & Seeger, 2005). Furthermore, unlike most previous cross‐sectional research (Chu et al., 2022), in this study, individuals’ mental distress was assessed at two time points, allowing to control for baseline levels and likely autoregressive effects.

Regarding sample size and respondent's characteristics (see Table 1), a total of 1162 individuals aged between 18 and 87 years participated in the two survey waves. Of those, 53% were male, and 47% were female. Their mean age was 53 years (*SD* =  13.98). In addition, 28% had the highest German education level, 35% had a medium, and 37% had a low education level.

**TABLE 1:** Distributions of respondents’ demographics.

	*t*_1_	*t*_2_
	(*n* = 1344)	(*n* = 1162)
**Gender**		
Female	48%	47%
Male	52%	53%
**Age**	52 (*SD* = 14.39)	53 (*SD* = 13.98)
18–29 years	9%	8%
30–39 years	13 %	12%
40–49 years	16%	15%
50–59 years	25%	25%
60 years and older	37%	39%
**Education level**		
Low	36%	37%
Medium	35%	35%
High	29%	28%

*Note*. *t*_1_: September 15–23, 2021; *t*_2_: December 9–20, 2021. Low education level: lowest German school degree (elementary school or lower secondary school diploma) or no school diploma; Medium education level: mid‐level secondary school diploma; High education level: higher secondary school diploma, university admission, or university graduation.

### 3.2 Measures

#### 3.2.1 Mental distress (*t*_1_ and *t*_2_)

To measure the respondents’ mental distress, their *depression levels* were assessed using the patient health questionnaire‐8 (PHQ‐8; Kroenke et al., 2009). The respondents indicated how often they experienced eight symptoms (e.g., “Little interest or pleasure in doing things” and “Feeling down, depressed, or hopeless”) over the previous 2 weeks (0  = *not at all*, 1 = *several days*, 2 = *more than half the days*, 3 = *nearly every day*). The items were summed to an index ranging from 0 to 24 (*M_t_*_1_ =  5.20, *SD_t_*_1_ =  5.73; *M_t_*_2_ =  5.51, *SD_t_*_2_ =  5.94), with higher values indicating higher levels of mental distress (see Table A1 and A2 for more details). A paired *t*‐test showed that depressive symptoms at *t*_2_ were slightly but significantly elevated compared to *t*_1_ (*t*(1,136)  =  −2.96, *p* =  0.001, *d *=  0.10).

#### 3.2.2 COVID‐19‐related information exposure (*t*_2_)

To measure COVID‐19‐related traditional news media exposure, the respondents indicated how often they used five common types of information sources in the German media landscape to obtain relevant information over the previous 4 weeks (0  = *never*, 5 = *several times a day*). Following theoretical considerations and previous research (Viehmann et al., 2021), *high‐quality news media exposure* included (1) public broadcasters, including their online services^1^; (2) national quality newspapers and magazines, including their online services; and (3) local and regional quality newspapers, including their online services (*M_t_*_2_ =  2.01, *SD_t_*_2_ =  1.21). *Low‐quality news media* *exposure* included (1) private broadcasters, including their online services, and (2) tabloids, including their online services (*M_t_*_2_ =  1.37, *SD_t_*_2_ =  1.20).

To assess *social media* *exposure* to COVID‐19‐related information, the respondents indicated how often they obtained such information from Facebook, Instagram, YouTube, and Twitter (0  = *never*, 5 = *several times a day*, *M_t_*_2_ =  0.76, *SD_t_*_2_ =  0.97), as these were among the most frequently used platforms across the German population (Beisch & Koch, 2021; Nielson et al., 2021). For an overview, see Table A3.

#### 3.2.3 Perceived threats (*t_2_*)

Based on the TSM (Lazarus, 1999) and contemporary related works (e.g., So, 2013), perceived threats of COVID‐19 were assessed using cognitive and emotional indicators (1 = *does not apply*, 5 = *fully applies*; see Table A4 for a detailed overview). For the cognitive indicators, perceived severity and susceptibility were measured, while the emotional indicators differed depending on the type of threat (e.g., So, 2013). *Perceived health threat* was measured with six items indicating (a) to what extent respondents perceived a serious health threat posed by the coronavirus in general (perceived severity) as well as (b) for their individual situation (perceived susceptibility), and (c) how much fear (relational emotion of perceiving a physical threat) they felt. The items included, for instance, “COVID‐19 is a serious health threat for the German population”, “COVID‐19 poses a health threat to me personally”, and “Fear of getting infected” *(M_t_*_2_ =  3.44, *SD_t_*_2_ =  1.01). *Perceived political threat* was measured using four items indicating (a) the extent to which the respondents perceived the consequences of the governmental protection measures as a threat to fundamental rights in general (perceived severity) as well as (b) within their personal lives (perceived susceptibility), and (c) how much anger they felt (relational emotion of perceiving a threat to personal freedom). The items included, for example, “The measures against the spread of COVID‐19 severely restrict the fundamental rights in Germany”, “Due to the measures taken to combat COVID‐19 in my area of residence, my fundamental rights are severely restricted”, and “Anger of being not allowed to do a lot of things that I usually do” (*M_t_*_2_ =  2.78, *SD_t_*_2_ =  1.14). Using principal component analysis, a two‐dimensional structure of perceived health and political threat was confirmed (Kaiser–Meyer–Olkin =  0.85, *p* < 0.001).

### 3.3 Data analysis procedure

First, bivariate correlations between media exposure, perceived threats, and depressive symptoms were calculated. Then, to analyze direct and indirect effects, a multiple mediation model^2^ was applied using the lavaan package in R (Rosseel, 2012). The model used depressive symptoms at *t*_2_ as the dependent variable, exposure to COVID‐19‐related information through the three information sources over the 4 weeks preceding at *t*_2_ as predictors^3^, and perceived health and political threat at *t*_2_ as potential mediators. To control for autoregression, the lagged version of the dependent variable (depressive symptoms at *t*_1_) was included in the model. In line with previous research, the model was further adjusted for potential individual differences in gender, age, education, and financial burden due to the pandemic (Houston et al., 2018; Stainback et al., 2020).

Using complete case analysis (*n* = 1116), the model showed an acceptable fit (Hu & Bentler, 1999): comparative fit index  =  0.998, Tucker–Lewis index  =  0.973, root mean square error approximation  =  0.035 with 90% confidence interval (CI) [0.000, 0.077], and standardized root mean square residual  =  0.007. The chi‐squared statistic for the used model was *χ* ^2^  =  4.666, *df* =  2, *p* = 0.097, whereas that for the baseline model was *χ* ^2^  =  1527.941, *df* = 30, *p* = 0.000.

## 4 RESULTS

Table 2 shows the bivariate correlations between COVID‐19‐related media exposure, perceived threats, and depressive symptoms at *t*_1_ and *t*_2_. All associations were significant, except for the relationships between social media exposure (*t*_2_) and perceived health threat (*t*_2_) and between low‐quality news media exposure (*t*_2_) and depressive symptoms (*t*_1_ and *t*_2_).

**TABLE 2:** Bivariate correlations between respondents’ COVID‐19 media exposure, perceived threats, and depressive symptoms.

		1	2	3	4	5	6
1	High‐quality media exposure (*t*_2_)						
2	Low‐quality media exposure (*t*_2_)	0.34^***^					
3	Social media exposure (*t*_2_)	0.10^**^	0.10^**^				
4	Perceived health threat (*t*_2_)	0.26^***^	0.16^***^	−0.03			
5	Perceived political threat (*t*_2_)	−0.14^***^	0.08^**^	0.12^***^	−0.27^***^		
6	Depressive symptoms (*t*_2_)	−0.10^***^	0.04	0.19^***^	0.12^***^	0.24^***^	
7	Depressive symptoms (*t*_1_)	−0.07^*^	0.04	0.17^***^	0.14^***^	0.20^***^	0.80^***^

*Note. t*_1_: September 15–23, 2021; *t*_2_: December 9–20, 2021. ^*^ *p *< 0.05, ^**^ *p* < 0.01, ^***^ *p* < 0.001.

Partially in contrast to correlation results, the mediation analysis neither revealed a significant direct effect of COVID‐19‐related low‐quality news media exposure (*β* = 0.015, *p* = 0.417) nor of high‐quality news media exposure (*β* = −0.023, *p* = 0.292) on depressive symptoms. Therefore, H1 was not supported. Social media use was also not significantly associated with depressive symptoms (*β* =  0.039, *p* =  0.052), thus indicating no difference in its effects compared to exposure to COVID‐19‐related information through traditional news media sources (RQ1).

**FIGURE 1:**
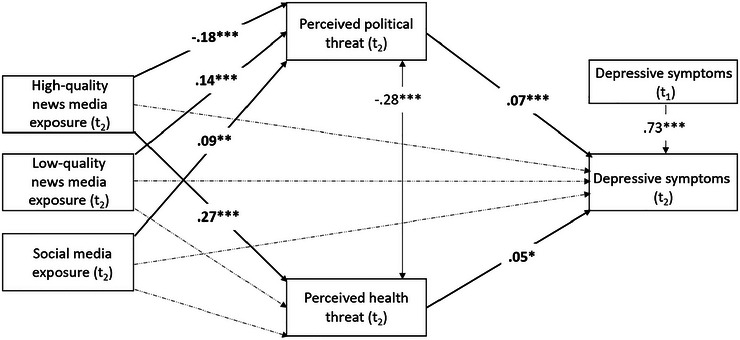
The figure shows the results of the mediation analysis (*n* = 1116). Values represent standardized beta coefficients. The covariates included in the model were age, gender, education, and financial burden due to COVID‐19. *t*_1_: September 15–23, 2021; *t*_2_: December 9–20, 2021. ^*^ *p* < 0.05, ^**^ *p* < 0.01, ^***^ *p* < 0.001. Indirect effect high‐quality media via perceived political threat: *β* = −0.013, 95% CI [−0.023, −0.006]. Indirect effect low‐quality media via perceived political threat: *β* = 0.010, 95% CI [0.004, 0.019]. Indirect effect social media via perceived political threat: *β* = 0.006, 95% CI [0.002, 0.014]. Indirect effect high‐quality media via perceived health threat: *β* = 0.013, 95% CI [0.002, 0.025]. Indirect effect low‐quality media via perceived health threat*: β* = 0.003, 95% CI [0.000, 0.008]. Indirect effect social media via perceived health threat: *β* = −0.003, 95% CI [−0.009, 0.000].

Furthermore, as shown in Figure 1, media exposure indirectly influenced depressive symptoms through perceived threat, but the pathways through which media exposure affected depressive symptoms depended on the types of information sources. More specifically, the more the respondents were exposed to pandemic‐related information through high‐quality news media, the greater their perceived health threat was (*β *= 0.268, *p* < 0.001), which, in turn, led to stronger depressive symptoms (*β* = 0.048, *p* = 0.023; indirect effect: *β* = 0.013, 95% CI [0.002, 0.025]).

In contrast, using low‐quality news media (indirect effect: *β* =  0.003, 95% CI [0.000, 0.008]) and social media platforms (indirect effect: *β* =  −0.003, 95% CI [−0.009, 0.000]) did not affect depressive symptoms through perception of health threat, but it did so through *perceived political threat*. Respondents with more frequent exposure to COVID‐19‐related information through low‐quality news media (*β* =  0.136, *p* < 0.001) and social media (*β* =  0.086, *p* =  0.007) were more concerned about the consequences of governmental restrictions for fundamental rights, leading to more severe depressive symptoms (*β* =  0.074, *p* < 0.001; indirect effect of low‐quality media: *β* =  0.010, 95% CI [0.004, 0.019], indirect effect of social media: *β* =  0.006, 95% CI [0.002, 0.0014]). Conversely, being exposed more frequently to COVID‐19‐related information through high‐quality news media was associated with lower perceived political threat (*β* =  −0.176, *p* < 0.001) and, thus, indirectly with milder depressive symptoms (indirect effect: *β* =  −0.013, 95% CI [−0.023, −0.006]).

Therefore, in the context of perceived health threat, the mediation hypothesis H2 was supported for high‐quality traditional news media exposure only. Regarding perceived political threat, the hypothesis was merely supported for low‐quality traditional news media exposure because high‐quality media exposure had, contrary to expectations, a negative indirect effect. Moreover, concerning RQ1, the (nonsignificant) indirect effects of COVID‐19 pandemic‐related social media exposure were significantly different from those of exposure through high‐quality traditional news media (relative indirect effects via perceived health and political threat *p* < 0.05), but similar to the (nonsignificant) indirect effects of being exposed to low‐quality traditional news media on depressive symptoms (relative indirect effects via perceived health and political threat *p* > 0.05). Thereby, it is noteworthy that the autoregressive effects of depressive symptoms were very strong (*β* =  0.73, *p* < 0.001), leaving little room for direct and indirect media effects on mental health and partially explaining the small effect sizes of all types of information sources.

## 5 DISCUSSION

The aim of this study was to examine mental health risks associated with the media's risk communication on public health crises, particularly focusing on the COVID‐19 pandemic. Specifically, it was sought to provide a comprehensive account of how pandemic‐related media exposure affects individuals’ depressive symptoms directly as well as indirectly mediated through perceived threat. For this purpose, the present study considered both perceived health and political threat as well as three different types of media sources (low‐quality traditional news media, high‐quality traditional news media, and social media platforms).

Contrary to expectations based on previous cross‐sectional research (Chu et al., 2022; Wu et al., 2021), this two‐wave panel survey study found no evidence that COVID‐19‐related media exposure directly affected individuals’ depressive symptoms, neither through traditional news media nor social media consumption. One possible reason for these partially contrasting findings compared to earlier (mediation) studies (e.g., Wang et al., 2021; Wu et al., 2021) could be that the present study accounted for highly autoregressive effects of prior depressive symptoms at *t*_1._ This suggests that previous cross‐sectional research potentially has overestimated the impact of media exposure. Supporting this assumption, a post hoc mediation analysis without controlling for autoregressive effects indicated a significant positive direct effect of social media exposure and a negative effect of high‐quality media exposure on depressive symptoms. Moreover, unlike prior research focusing on mental health‐related media effects during the initial stage of the COVID‐19 pandemic (e.g., Wu et al., 2021), the present study is based on data collected later in the crisis—specifically, during the so‐called maintenance phase (Reynolds & Seeger, 2005). Therefore, an additional explanation could be that crisis risk communication by traditional news media (Hase & Engelke, 2022; Reinemann et al., 2024) and on social media platforms (Iglesias‐Sánchez et al., 2020) evolved over the course of the pandemic in such a way that the rapid spread of information and content containing potentially distressing themes, uncertainty, and fear‐inducing language decreased. Also, it may be possible that individuals became desensitized to prolonged pandemic‐related content, and as a result, their mental health was no longer directly affected by media exposure. Specifically, the disaster literature proposes that stress responses can diminish with repeated exposure to similar or identical stressors (Garfin et al., 2015), such as ongoing health crisis‐related media information. While these findings highlight the importance of longitudinal designs for examining mental health risks associated with communication on public risks and crises, they also underscore the need for more multimethod studies. In particular, analyzing both the content of disseminated media information (through content analyses) and its effects (through surveys) across different stages of a health crisis is essential to gain a comprehensive understanding of potentially time‐varying relationships.

Another major aim was to examine the mediating role of perceived threats, drawing on the TSM (Lazarus, 1999), works from the field of health risk communication (e.g., So, 2013), and literature on the DSMM (Houston et al., 2018). First, the findings indicate that, instead of direct influences, traditional news media and social media effects on depressive symptoms were fully mediated by perceived health threat (regarding the threat posed by the virus) and/or perceived political threat (regarding personal freedom). Together with extant mediational studies (e.g., Wu et al., 2021), this suggests that the media's impact on individuals’ threat perceptions is indeed a significant mechanism linking media consumption and mental health during times of public health crisis. From a theoretical perspective, this study thus supports the assumption of the TSM that threat perceptions play a crucial role in the development of mental distress and shows that this also applies to public health crisis‐related threats. Moreover, the study's findings enrich the TSM by identifying *media*‐related stressors—including exposure to public health crisis risk communication through traditional news media and social media platforms—that shape specific threat perceptions as relevant risk factors for impaired mental health. In light of the TSM and research on health crisis risk communication (e.g., Friemel & Gerber, 2023; Hase & Engelke, 2022; Klemm et al., 2016), this result might be explained by the general predominance of threat‐related content over coping messages in the media. In addition, the findings are consistent with the sequential pathways proposed by the DSMM (Valkenburg & Peter, 2013) and contribute to the related crisis media effects literature: While Houston et al. (2018) demonstrated indirect, second‐order media effects on psychological outcomes in disaster situations (e.g., natural disasters), the present study shows that these mechanisms also extend to a major health crisis, uncovering threat perceptions as important media effect mediators in this context.

Second, the present study points out the importance of considering distinct types of both perceived threats and information sources in (health) crisis risk communication research on mental distress and the DSMM, as exposure to various media can lead to differing effects. Specifically, perceived health threat mediated only the relationship between mental health and the use of COVID‐19‐related information through high‐quality news media. This indicates that individuals exposed to crisis risk information from reliable sources tended to perceive greater physical health threat, which in turn negatively affected their mental health. In contrast, no mediating effects of health threat perception were observed for less reliable low‐quality news media (commercial broadcasting services and tabloid press) or social media platforms. Instead, exposure to these two types of media increased depressive symptoms by amplifying perceived political threat, whereas using high‐quality media even had a decreasing indirect effect in this context. Thus, this study suggests that the distressing effects of different types of information sources are exerted through distinct pathways. While these results partly contrast with earlier studies that exclusively reported significant *distressing* indirect effects of (social) media exposure via perceived threats (e.g., Wang et al., 2021), they align with research showing differential media effects on other COVID‐19‐related outcomes (e.g., support for protective measures; Frissen et al., 2020) and other social issues (e.g., immigration; Jacobs et al., 2016). The present study therefore advances this line of research by demonstrating that differential (indirect) media effects also apply to mental distress.

A possible explanation for the study results may be that the three types of information sources differ in how they communicate public health crises: A recent content analysis of German news media coverage (Reinemann et al., 2024) found that tabloid newspapers more frequently emphasized the impact of COVID‐19 measures on personal freedom than quality media, which clearly focused on health threats and safety. Over time, issues related to personal freedom generally gained prominence in the later stages of the pandemic, while health threats and safety largely dominated media coverage during its initial phase. In addition, research on previous health crises (Rossmann et al., 2018) indicates that the quality media tend to report on protective measures more often than low‐quality news media—a finding recently supported by studies on COVID‐19‐related television coverage in Germany (e.g., Wiedicke et al., 2024). Taken together, these content differences and shifts over time may not only explain why this study unexpectedly found distressing effects of low‐quality media exposure exclusively through perceived political threat—not health threat—during the maintenance phase of the pandemic. They could also account for the surprisingly calming effect of using high‐quality news media on perceived political threat and, thus, on individuals’ depressive symptoms. To gain deeper insights here, future content analyses should specifically examine whether COVID‐19‐related information in low‐quality traditional news media might have been linked to political threats in a negative, anger‐inducing manner more often during the maintenance phase than in high‐quality media. And, conversely, whether high‐quality traditional news media have focused more on fear‐inducing threats for personal and public health, as well as on the efficacy of protective measures. Thereby, researchers could also explore whether the political orientation of media outlets (e.g., Reinemann et al., 2024) contributes to potentially distressing public health crisis risk communication patterns.

Similar to low‐quality traditional news media, German social media discussions frequently addressed the impacts of COVID‐19 on social life, including concerns about restrictions on fundamental rights, but also the spread of health misinformation (Wahidie et al., 2021). Unlike traditional news media information, however, social media communication during health crises is characterized by negative emotional expressions (e.g., anger and sadness), often embedded in highly engaging and, therefore, particularly distressing personal stories (Iglesias‐Sánchez et al., 2020; Seo, 2021). While the observed indirect effect of COVID‐19‐related social media exposure on mental distress—mediated by perceived political threat—seems plausible, it is surprising that it was as small as the distressing effects of traditional news media sources, especially when compared to high‐quality media. One possible explanation could be the role of selective social media use, amplified by personalized algorithms (Taylor & Choi, 2024; Valkenburg et al., 2022): Some users might have been continuously exposed to exclusively distressing content, whereas others encountered more supportive messages, leading to small overall effect sizes. Longitudinal experience sampling studies could help examine this potential heterogeneity in social media effects (Beyens et al., 2020).

In summary, exposure to crisis‐related information through social media platforms and low‐quality traditional news media mainly had adverse consequences. In contrast, understanding the impacts of health crisis risk communication by high‐quality media appears more complex, posing risks to mental health while also exhibiting health‐promoting potential.

Beyond these insights, the study also has broader implications for the field of health crisis risk communication: Literature related to the E‐EPPM (e.g., Friemel & Geber, 2023; So, 2013) suggests that fear‐inducing threat information is essential during health crises because it increases recipients’ perceived health threat, which can subsequently encourage protective behaviors if coping appraisals are sufficient. However, this study indicates that health threat perceptions also mediate the negative mental health effects of media exposure. Thus, this underscores the need for future studies to address both negative psychological and positive behavioral outcomes when designing and evaluating strategies for communicating major health risks. Moreover, given the observed differential media effects across distinct types of perceived threat and the previous literature's focus on health threat perceptions, such studies could also explore whether including political threat perceptions enhances our understanding of (inadequate) protective behaviors. In conclusion, investigating the relationships between individuals’ use of diverse information sources, their various threat perceptions (such as health and political), and both behavioral and mental health outcomes may be vital for advancing health crisis communication research.

## 6 LIMITATIONS AND FUTURE RESEARCH

This study has several limitations. Methodologically, these include the focus on measuring perceived threat, while individual coping appraisals were not investigated. Although coping appraisals may be partially reflected in perceived threat (So et al., 2016), future studies should explore how they are influenced by the use of the different media types and how they interact with threat perceptions and mental distress. Moreover, this study examined overall social media exposure across several platforms, without differentiating between exposure to user‐generated content and posts from traditional news media or other reliable sources, such as health experts or policymakers. Future research could therefore distinguish between social media content shared by different sources. Thereby, researchers could also consider additional social media platforms, such as TikTok, which has recently become popular particularly among younger users (Beisch & Koch, 2021), as well as other social media types like messaging services (e.g., Telegram). All this could provide a more comprehensive understanding of the role of media in shaping mental health‐relevant perceptions during health crises.

In addition, this study has methodological limitations related to its two‐wave panel survey design: First, the design did not fully permit the establishment of causality, as it does not allow for the accurate separation of intraindividual changes from between‐person differences (Thomas et al., 2021). Second, it did not allow for the examination of various potential longitudinal developments, such as (de)sensitization, reciprocal effects (e.g., between perceived threats and use of information sources), or even transactional dynamics (e.g., reinforcing cycle). Longer‐term panel studies are consequently necessary to explore different possible dynamic interrelationships between media exposure, perceived threat, and mental health across the different communication stages of a prolonged health crisis.

From a theoretical perspective, the findings are not generalizable to other types of (multifaceted) crises. To further advance our knowledge of crisis‐related media effects (Houston et al., 2018), researchers could examine the mental health risks of media communication and threat perceptions as mediators of media effects in contexts such as climate change or armed conflicts. This would help determine whether the observed mechanisms are specific to health crises or applicable across crisis contexts. Finally, the effect sizes of the observed indirect effects were relatively small. Although smaller effect sizes are common in longitudinal designs compared to cross‐sectional surveys, it is possible that other unobserved (moderating) factors played a role. Therefore, future studies should also consider nonmedia variables, such as individuals’ political orientation (Zhou et al., 2023) or perceived social support (Fruehwirth et al., 2024), as these could influence both media use and its impact on mental health during public health crises.

## 7 PRACTICAL IMPLICATIONS

With the identification of perceived threats as mediators between media exposure and mental health, the results of this study also indicate major practical challenges in the risk communication of public health crises through traditional news media and on social media platforms. Given that effectively communicating health threats is a driving force of compliance with protective measures during epidemics or pandemics (e.g., B. Chan et al., 2024), the question arises as to how to keep recipients of high‐quality news media informed and raise awareness regarding health threats without negatively affecting their mental health. Therefore, a possible conciliatory strategy may be to balance fear‐inducing threat information with realistic, hope‐inducing coping messages. Following the TSM (Lazarus, 1999) and health risk communication literature (e.g., Friemel & Geber, 2023; So, 2013), coping appraisals and information are vital for both mental health and adequate health risk perception and protection behavior. Coping messages could include information on effective individual measures and institutional crisis management (Friemel & Geber, 2023) or potential paths forward. Moreover, journalists could report about positive people‐centered stories of coping with pandemic‐related threats and challenges or explicitly inform about potential strategies and support services to prevent or deal with mental distress. All such messages may promote hope‐inducing feelings of self‐ and response‐efficacy (Petersen et al., 2022; So et al., 2016), thereby potentially mitigating distressing effects of media exposure.

With regards to low‐quality traditional news media and social media platforms, the trade‐off between the right to free expression, including criticism and anger against protective measures, and negative psychological effects represents an additional challenge. However, it may be unrealistic to expect that journalists from market‐driven media outlets, respectively, private users embrace the proposed health crisis risk communication strategy. Several approaches might be helpful to minimize the negative impact of such media: For instance, politicians and public authorities could make use of their power and integrate both threat and coping information in their press releases and strategic communications via traditional news media and social media platforms. Among these public actors, health communication professionals—particularly public health authorities and related institutions—should strengthen their presence on social media and provide guidance to users on how to manage distressing content (e.g., anger‐inducing posts). Finally, at the institutional level, policymakers could advocate for media literacy training to empower individuals to cope with unavoidable crisis information effectively themselves. Nevertheless, future research in this area is needed to gain deeper insights into which message characteristics actually shape distressing threat perceptions, especially political threat, and how risk messages about a health crisis can be communicated sensitively in practice.

## 8 CONCLUSIONS

By providing a better understanding of the complex interrelationship between media exposure, perceived threats, and mental health during public health crises, this panel study contributes to the field of risk communication research in several ways. In the context of the COVID‐19 crisis, this study emphasizes the importance of considering not only perceived health threat in examining pandemic‐related risks. Media exposure to public health crises can indirectly affect mental health through political threat perceptions (e.g., regarding personal freedom) as well, and thus they should also be considered in risk communication research on public health crises in general and as risk factor for impaired mental health in particular. Furthermore, this study highlights the need to additionally distinguish between types of media sources (e.g., high‐ and low‐quality traditional news media and social media platforms), as they influenced perceived threats, and, in consequence, depressive symptoms differently. Researchers, media practitioners, as well as health professionals should be aware of the potentially dual impact of risk communication on public health crises and jointly develop strategies for mitigating adverse outcomes and enhancing mental health‐promoting effects across different stages of communication.

## CONFLICT OF INTEREST STATEMENT

The author declares no conflicts of interest.

## ETHICAL APPROVAL

I confirm that this study meets ethical guidelines to the requirements of the German Psychology Society. This research obtained approval from the ethic committee of the Technical University Ilmenau (approval number: 2022‐02‐178_FoA‐Schal.1).

## Data Availability

The data we used has not been published so far. If needed the data can be made available.

## Footnotes

1 The online services of the news media sources primarily refer to their websites and apps. Although news outlets also share online information on social media platforms, a study by Mellado et al. (2021) found that only 6% of analyzed German social media posts about the pandemic originated from news media sources.

2 Following Hayes (2018), bootstrapping with 5000 iterations was used to compute bias corrected 95% CIs for the indirect effects.

3 Given that the measurement of media exposure relates to the 4 weeks preceding, and thus already includes a time lag, it was decided to specify media exposure at *t*_2_ and not at *t*_1_ as the predictor of perceived threats and depressive symptoms.

## 1

**TABLE A1:** Measurement of respondents’ depression level.

**Question**	Over the last 2 weeks, how often have you been bothered by any of the following problems?
**Scale**	0 = *not at all*, 1 = *several days*, 2 = *more than half the days*, 3 = *nearly every day*
**Items**	Little interest or pleasure in doing things
	Feeling down, depressed, or hopeless
	Trouble falling or staying asleep, or sleeping too much
	Feeling tired or having little energy
	Poor appetite or overeating
	Feeling bad about yourself‐or that you are a failure or have let yourself or your family down
	Trouble concentrating on things
	Thoughts that life is not worth living
**Index and Reliability**	Sum index ranging from 0‐24 Cronbach's Alpha (*α*) *t_1_* and *t_2_* = 0.93

**TABLE A2:** Distribution of respondents’ depression level.

	*t_1_*	*t_2_*
*M*	5.20	5.51
*SD*	5.73	5.94
none (0‐4)	56%	56%
mild (5‐9)	24%	24%
moderate (10‐14)	11%	10%
moderately severe (15‐19)	6%	8%
severe (20‐24)	4%	4%

*Note*. *t_1_*: September 15–23, 2021; *t_2_*: December 9–20, 2021. Depression level can range between 0 and 24, whereby higher values indicate higher levels of mental distress.

**TABLE A3:** Measurement of respondents’ COVID‐19 related media exposure.

Variable	Items	*α* / *r*	*M*	*SD*
**High‐quality news media exposure** (*t_2_*, mean index)	Public broadcasting, such as ARD or ZDF, including their online services National newspaper and magazines, including their online services Local and regional newspapers, including their online services	0.58 (*α*)	2.01	1.21
**Low‐quality news media exposure** (*t_2_*, mean index)	Private Broadcasting, such as RTL or Sat.1, including their online services Tabloid newspapers, such as BILD, including their online services	0.24 (*r*)	1.37	1.20
**Social media exposure** (*t_2_*, mean index)	Facebook Instagram YouTube Twitter	0.67 (*α*)	0.76	0.97

*Note*. Question: In the last 4 weeks, how often have you received information about COVID‐19 via these sources? (0 = *never*, 1 = *less frequently than once a week*, 2 = *about once a week* 3 = *several times a week*, 4 = *daily*, 5 = *several times a day*.) Higher values indicate more frequent COVID‐19‐related (social) media exposure. *t_2_*: December 9–20, 2021

**TABLE A4:** Measurements of respondents’ perceived health and political threat.

Variable	Items	*α*	*M*	*SD*
**Perceived health threat** (*t_2_*, mean index)	COVID‐19 is a health threat for me personally. COVID‐19 is a health threat for people close to me. COVID‐19 is a serious health threat for the German population. Fear of getting infected Worry for people close to me I am afraid that many people in Germany will become infected.	0.91	3.44	1.01
**Perceived political threat** (*t_2_*, mean index)	Due to the measures taken to combat COVID‐19 in my area of residence, my fundamental rights are severely restricted. The measures against the spread of COVID‐19 severely restrict the fundamental rights in Germany. Anger of being not allowed to do a lot of things that I usually do I am angry that people in Germany are not allowed to do many things that they usually do.	0.87	2.78	1.14

*Note*. Question: In your opinion, to what extent do the following statements apply or not apply? (1 = *does not apply at all*, 5 = *fully applies*). Higher values indicate higher levels of perceived health and political threat. *t_2_*: December 9–20, 2021.
